# Analysis of the Physico-Chemical, Mechanical and Biological Properties of Crosslinked Type-I Collagen from Horse Tendon: Towards the Development of Ideal Scaffolding Material for Urethral Regeneration

**DOI:** 10.3390/ma14247648

**Published:** 2021-12-12

**Authors:** Nunzia Gallo, Maria Lucia Natali, Claudia Curci, Angela Picerno, Anna Gallone, Marco Vulpi, Antonio Vitarelli, Pasquale Ditonno, Mariafrancesca Cascione, Fabio Sallustio, Rosaria Rinaldi, Alessandro Sannino, Luca Salvatore

**Affiliations:** 1 Department of Engineering for Innovation, University of Salento, 73100 Lecce, Italy; m.natali@typeone.it (M.L.N.); alessandro.sannino@unisalento.it (A.S.); luca.salvatore@unisalento.it (L.S.); 2 Typeone Biomaterials, Via Vittorio Veneto 64/C, 73036 Muro Leccese, Italy; 3 Department of Basic Medical Sciences, Neurosciences and Sense Organs, University of Bari “Aldo Moro”, 70124 Bari, Italy; claudiacurci@gmail.com (C.C.); anna.gallone@uniba.it (A.G.); 4 Nephrology Unit, Department of Emergency and Organ Transplantation, University of Bari “Aldo Moro”, 70124 Bari, Italy; angelapicerno89@gmail.com; 5 Urology and Andrology Unit, Department of Emergency and Organ Transplant, University of Bari “Aldo Moro”, 70124 Bari, Italy; vulpimarco@gmail.com (M.V.); antoniovitarelli@hotmail.com (A.V.); pasquale.ditonno@uniba.it (P.D.); 6 Department of Mathematics and Physics “Ennio De Giorgi”, University of Salento, 73100 Lecce, Italy; mariafrancesca.cascione@unisalento.it (M.C.); ross.rinaldi@unisalento.it (R.R.); 7 Department of Interdisciplinary Medicine, University of Bari “Aldo Moro”, 70124 Bari, Italy; fabio.sallustio@uniba.it

**Keywords:** type I collagen, urethra, stem cell, scaffold, tissue engineering

## Abstract

Urethral stenosis is a pathological condition that consists in the narrowing of the urethral lumen because of the formation of scar tissue. Unfortunately, none of the current surgical approaches represent an optimal solution because of the high stricture recurrence rate. In this context, we preliminarily explored the potential of an insoluble type-I collagen from horse tendon as scaffolding material for the development of innovative devices for the regeneration of injured urethral tracts. Non-porous collagen-based substrates were produced and optimized, in terms of crosslinking density of the macromolecular structure, to either provide mechanical properties compliant with the urinary tract physiological stress and better sustain tissue regeneration. The effect of the adopted crosslinking strategy on the protein integrity and on the substrate physical–chemical, mechanical and biological properties was investigated in comparison with a decellularized matrix from porcine small intestinal submucosa (SIS patch), an extensively used xenograft licensed for clinical use in urology. The optimized production protocols allowed the preservation of the type I collagen native structure and the realization of a substrate with appealing end-use properties. The biological response, preliminarily investigated by immunofluorescence experiments on human adult renal stem/progenitor cells until 28 days, showed the formation of a stem-cell monolayer within 14 days and the onset of spheroids within 28 days. These results suggested the great potential of the collagen-based material for the development of scaffolds for urethral plate regeneration and for in vitro cellular studies.

## 1. Introduction

Urethral stenosis is a pathological condition that affects approximately 0.6–1.0% of the world male population, significantly affecting their quality of life [1,2,3,4]. It is a urinary tract disease that consists in the narrowing of the urethral lumen due to abnormal formation of scar tissue mainly for iatrogenic, idiopathic, inflammatory and traumatic reasons [5,6]. Typical symptoms include weak urinary stream, straining to void, urinary hesitancy, incomplete emptying and nocturia [7]. Other signs and symptoms, such as genitourinary pain, urinary tract infection, ejaculatory dysfunction, urethral discharge, and hematuria may also occur [8].

The adopted surgical approach strictly depends on the stricture length. In particular, strictures shorter than 1–2 cm are treated by urethrotomy (except for anterior urethra) or end-to-end anastomosis, with approximately 80% success [9]. In the case of defects longer than 2 cm, augmentation urethroplasty is the preferred approach [10]. Endoscopic dilatation has a success rate of 50–60% for short strictures, a percentage that decreases at approximately 20% in the case of strictures longer than 2 cm [1]. The reason why urethral endoscopic dilation is not effective is that it implies the rupture of the urethral mucosa and subsequent urine diffusion in the created defect, which further nourishes an inflammatory response resulting in the formation of scar tissue [11]. Anastomotic urethroplasty, which has the highest success rate (93%) [11], is burdened by the risk of excessive tension on the penis, which may cause chordee or curvature and may impact erectile function [12]. Regarding urethroplasty, onlay augmentation is performed by mean of autografts from prepuce skin, buccal or sublingual mucosa, among which buccal mucosa is the most effective [13,14]. Augmentation urethroplasty with buccal mucosa graft is the gold standard in stricture treatment (most common donor sites are also cheek, labial and sublingual mucosa). However, it is affected by issues such as donor site morbidity (i.e., pain, infection, discomfort, numbness) [15], change of the donor site in case of recurrence (e.g., left cheek, right cheek and the labial), hindrance in buccal mucosa collection in cases of ongoing oral infection, restricted mouth opening or extension, previous mouth or tongue surgery [16]. Thus, some decellularized xenografts have been tested for augmentation urethroplasty, among which the most promising was a decellularized matrix from the suine small intestinal submucosa (SIS patch). Although urethroplasty with SIS patch is a promising approach, it is not resolutive since it represents a 20–25% stricture recurrence rate within 1–5 years of follow-up [17].

The absence of resolutive solutions among all current approaches due to the not negligible degrees of stricture recurrence rate necessitates the development of new strategies. In the last few decades, many attempts were made to solve this clinical problem [9,11,18,19,20]. Among them, tissue engineering-based approaches showed promising results [19,21,22,23,24]. In this method, the fundamental role of the scaffold is to provide a temporary structural support for cell adhesion and proliferation in order to promote new tissue development [21,22]. The most promising biomaterials employed for the manufacture of tissue engineering-based devices are components of the extracellular matrix (ECM), such as collagen [23,24], hyaluronic acid [25], elastin [26], and glycosaminoglycans [27]. Among them, type I collagen is widely used for the manufacture of tissue-engineering medical products (TEMPs) thanks to its well-known structural properties and biological activity [23,28,29,30].

In this work, an insoluble type-I collagen isolated from horse tendon was used for the preparation of the substrates under study [31]. This mammalian source of collagen, compared to analogues bovine and swine [32,33,34], was chosen for its freedom from issues such zoonosis transmission risks (e.g., bovine spongiform encephalopathy and immune-response triggering [35,36,37,38,39], which affect other animal extraction sources [36,40]. Although it is not well known, it should be mentioned that equids are exposed to alphaviral equine encephalomyelitis (AEE), a mosquito-borne zoonotic infection that only occasionally spills over in humans [41,42]. The low occurrence of encephalitis cases and the very low AEE-induced mortality rate lead to the consideration of equine by-products as zoonosis-free extraction sources for medical-grade collagen [35]. Moreover, the high hierarchical organization of collagen fibers in tendon and their strict packing are the reasons why devices manufactured with reconstituted native horse-tendon collagen are intrinsically more resistant to degradation and mechanical stress [40,43]. These properties are particular appealing for the development of devices for urethral regeneration, where scaffolds need to have mechanical properties compliant with urinary tract physiological stress.

In this study, horse collagen was processed in the form of ‘film-like’ solid substrates, following optimized processing conditions, in order to develop a formulation able to sustain tissue regeneration better than the SIS patch, currently the only licensed xenograft for clinical use [31,43]. The collagen substrates were thus appropriately crosslinked, sterilized, and characterized from a physical–chemical, mechanical and biological point of view in comparison with the SIS patch. Particular attention was paid to the preservation of the fiber structure, a key aspect that influences not only bioengineering parameters but also the cell-biomaterial interaction, since the nanometric fibril organization is recognized by cells as a guide for cell growth and migration during the remodeling phase of the healing process [24,44,45,46]. To this end, zero-length crosslinking methods were employed to improve scaffold mechanical performance and degradation resistance without affecting the collagen’s three-dimensional conformation-related bioactivity and degradation by-product nature.

First of all, the effect of the adopted crosslinking strategy on the protein integrity was investigated on polyacrylamide gel electrophoresis in the presence of sodium dodecyl sulphate (SDS-PAGE). The crosslinking degree was examined in terms of residual free amine groups by means of 2,4,6-trinitro-benzene-sulfonic acid (TNBS) test, and in terms of elastically effective crosslink density by means of stress-relaxation tensile test. Functional surface groups were investigated by Fourier Transform Infrared (FT-IR). Thermal properties were assessed by means of differential scanning calorimetry (DSC). The enzymatic resistance was determined by means of in vitro collagenase digestion. Wettability was investigated by swelling degree and contact angle measurements. Surface modification induced by the crosslinking strategy was investigated by atomic force microscopy (AFM). The substrate suitability in terms of mechanical properties was examined through tensile and suture retention tests. Finally, the preliminary in vitro biological response was investigated by human Adult Renal Stem/Progenitor Cells (ARPCs) seeding and immunostaining up to 28 days.

## 2. Materials and Methods

### 2.1. Materials

Insoluble fibrillar type I collagen from equine tendon in dry flake form was provided by Typeone Biomaterials Srl (Lecce, Italy). The SIS patch from Cook Biotech Inc (West Lafayette, IN, USA) was kindly provided by Polyclinic of Bari (Bari, Italy). Distilled water was obtained from Millipore Milli-U10 water purification facility from Merck KGaA (Darmstadt, Germany). Standard protein markers of precise molecular weights ranging from 10 to 250 kDa were provided by Bio-Rad Laboratories Inc (Hercules, CA, USA). Mouse anti-human β-Actin mAb was purchased from Sigma-Aldrich (Milan, Italy) while mouse anti-human CD133/2 mAb (clone AC1339) was purchased from Miltenyi Biotec (Bergisch Gladbach, Germany). The secondary antibodies Alexa Fluor 488 goat anti-mouse IgG and Alexa Fluor 555 goat anti-mouse IgG were purchased by Invitrogen, Life Technologies (Waltham, MA, USA). All other chemicals used were of analytical grade and purchased by Sigma-Aldrich (St. Louis, MO, USA).

### 2.2. Collagen Substrates Preparation

Non-porous substrates in the form of thin foil or ‘film’ were synthetized by air-drying following a previously optimized synthesis protocol [31]. Briefly, type I collagen was suspended in 0.5 M acetic acid, at a concentration of 10 mg/mL for 2 h, homogenized at 8000 rpm for 20 min and degassed under vacuum [43]. During the mixing process the suspension was refrigerated (4–10 °C) in order to avoid the denaturation of collagen fibers [47]. After slurry casting in Petri dishes, air-drying took place in a laminar flow hood for 72 h at room temperature [31].

Following fabrication, collagen films were exposed to dry heat (DHT: 121 °C, 72 h, *p* < 100 mTorr) [48] and then by incubated in a 1-etil-3-(3-dimetilaminopropil)-carbodiimide (EDC) and N-hydroxysuccinimide (NHS) crosslinking bath (14.0 mM EDC, 5.5 mM NHS, 2 h, room temperature) [49,50,51]. All crosslinked samples were washed three times in distilled water for 10 min in order to remove any residues of unreacted chemicals, and air-dried again. Lastly, the DHT-treated films and the double crosslinked films (DHT/EDC) were sterilized by means of dry heat at 160 °C for 2 h, under vacuum (*p* < 100 mTorr) [52].

### 2.3. Composition

The SDS-PAGE is a commonly employed analytical technique to analyze protein extracts. In particular, it was performed to assess both the collagen-based substrate protein integrity after crosslinking and sterilization treatments and SIS patch protein composition. The Mini-Protean Tetra Cell (Bio-Rad Laboratories Inc., Hercules, CA, USA) electrophoresis system on acrylamide gel was used for the analysis. Hand-cast gels (5% stacking gel, 10% resolving gel) were prepared using acrylamide/bisacrylamide solution with a ratio of 37.5:1 [53]. Samples of approximately 10 mg were minced, resuspended in 1 mL of 2 M HCl and then ground. Then a reducing solution composed of Laemmli buffer (62.5 mm Tris-HCl pH 6.8, 10% glycerol, 2% SDS, 0.01% bromophenol blue, 5% β-mercaptoethanol) and 2 M Urea was added to each sample. The protein band pattern of native type I collagen before any treatment was used as control to provide an example of protein integrity and purity [31]. Thus, a small amount of the prepared collagen gel (60 mg) was dissolved in 0.1 mL of reducing solution [31,54,55,56]. All samples were heat treated at 50 °C for 1 h, centrifuged for 1 min at 10,000× *g* and loaded in the electrophoresis wells.

The SDS-PAGE run was carried out at 70 V for approximately 10 min in the stacking gel and at 120 V for approximately 2 h within the resolving gel. After electrophoresis, the gel was Coomassie stained. Briefly, the gel was washed twice by soaking it in 100 mL of double distilled water, subjecting it to max power microwaves for 30 s and placing it on a shaker for 5 min. Then the gel was revealed by means of a Coomassie staining solution made of 0.008% Coomassie Blue R-250 and 0.036 M chloridric acid. After 10 s max power microwaves, the gel was gently shaken for 2 h, destained in double-distilled water and acquired [57].

### 2.4. Crosslinking Degree

The substrate crosslinking degree was evaluated by means of both the free amine group content and the elastically effective crosslinking density.

The content of the residual free primary amine groups of the DHT and the DHT/EDC films was determined using a TNBS assay following a protocol optimized for collagen-based substrates [48,52,58]. Samples of approximately 3 mg were hydrated for 30 min in 0.5 mL of 4% (*w*/*v*) NaHCO_3_ solution. Then each sample received an additional 0.5 mL of a freshly prepared solution of 0.05% (*w*/*v*) TNBS, was hermetically closed and was heated at 40 °C for 2 h. After that, 1.5 mL of 6 M HCl was added, and samples were hydrolyzed at 60 °C for 90 min. The reaction mixture was then diluted with 2.5 mL of distilled water and cooled down to room temperature. Finally, the absorbance at 320 nm was measured using a UV Spectrophotometer. Controls were prepared using the same procedure, except that HCl was added prior to introducing the TNBS solution to prohibit any reaction of the TNBS with the amine groups. The absorbance of the control samples was subtracted from each sample absorbance. The calibration line was obtained from the correlation of the absorbance with the concentration of several dilutions of a 0.1 mg/mL (*w*/*v*) of glycine in 4% (*w*/*v*) NaHCO_3_ solution. Three replicates were performed for each determination and the number of amino groups per 1000 residues calculated [58,59,60]. The as-produced dry film (uncross-linked) was assumed to contain 100% of the available free amine groups, and this value was used to calculate the crosslinking degree as the percentage of remaining free amine groups after the crosslinking treatment [61].

The crosslinking degree of a polymeric network is defined as the density of junctions joining the macromolecular chains into a matrix. The theory of rubber elasticity, which relates the degree of crosslinking of a rubber-like crosslinked polymer to its macroscopic mechanical properties, allows direct estimation of the concentration of all chemically crosslinked polymer segments [49,62]. Thus, samples (SIS patch, DHT and DHT/EDC films) were hydrated and denatured to be turned into rubber-like materials by soaking them in 0.01 M PBS at 80 °C for 2 min [48]. Then, samples were hydrated for 1 h in PBS and subjected to a stress-relaxation tensile test by means of a ZwickiLine universal testing machine (Zwick/Roell, Ulm, Germany) equipped with a loading cell of 1 kN and a bath chamber (5% deformation, 180 s dwelling time, 5 steps) [47]. According to the theory of rubber elasticity (Equation (1)), the obtained data from stress-relaxation tests were elaborated to calculate the shear modulus *G*, which is directly proportional to the elastically effective crosslink density *ρ* [63].

(1) $$\sigma =RT\rho V^{1/3}\left(\alpha -\frac{1}{\alpha^{2}}\right)=G\left(\alpha -\frac{1}{\alpha^{2}}\right)$$

where *σ* is the stress, *R* is the universal gas constant, *T* is the absolute temperature, *V* is the polymer volume fraction in the swollen state, α = L/L_i_ is the deformation ratio (with L the actual thickness of the deformed sample and L_i_ the initial thickness of the swollen sample (α > 1 for tensile), and *G* is the shear modulus of the swollen polymer. The test was performed in triplicate for each sample type.

### 2.5. Secondary Structure

FT-IR was performed by means of PerkinElmer Spectrum One IR spectrometer in ATR mode (Waltham, MA, USA) to achieve structural information on samples at the molecular level. Absorption spectra of DHT, DHT/EDC and SIS patch were recorded in the range 1800–800 cm^−1^ at a resolution of 4 cm^−1^, smoothed according to the Savitsky–Golay method and analyzed by means of the Origin software from OriginLab Corporation (Northampton, MA, USA). Each spectrum was collected as the average of 64 scans.

### 2.6. Swelling Degree

The substrates’ ability to retain water was gravimetrically evaluated. Dry samples of SIS patch, DHT and DHT/EDC films of approximately 1 × 1 cm were accurately weighted and soaked in 1 mL of 0.01 M phosphate-buffered saline (PBS). After the gentle removal of excess surface water with filter paper, the wet samples were weighed again at fixed time points. The experiment was performed in triplicate for each sample type. The % swelling degree (*SD*) was calculated by the following equation [64]:

(2) $$SD\%=\frac{W_{w}-W_{d}}{W_{d}}\;\cdot \;100$$

where *W_w_* is the sample wet weight at each fixed time point and *W_d_* is the initial dry weight.

### 2.7. Thermal Stability

DSC allows measurement of the protein denaturation temperature (Td) and thus the stability of the substrate in correlation to the crosslinking treatment [65]. Thermograms of SIS patch, DHT and DHT/EDC films were obtained using a Q2000 Series DSC (TA Instruments, New Castle, DE, USA). Samples were accurately weighted (5–10 mg) into aluminum pans, hermetically sealed and scanned over, with a first static step at 5 °C for 5 min and a second dynamic step from 5 °C to 80 °C at a scanning rate of 5 °C/min in inert nitrogen atmosphere (50 mL min^−1^) [52]. An empty aluminum pan was used as reference probe. Collagen denaturation temperature was recorded as the peak of the endothermic event and the enthalpy required for the transition was estimated as the area under the peak [66]. Each sample type was run in triplicate.

### 2.8. Degradation Resistance

The in vivo half-life of the thus-produced substrates was in vitro estimated with an enzymatic degradation test. Briefly, samples of approximately 10 × 10 mm were cut, weighted (W0) and incubated at 37 °C in 1 mL of a solution of 0.01 M phosphate-buffered saline (PBS), 0.34 mM CaCl_2_ and 0.1 mg/mL collagenase from Clostridium histolyticum [31]. At fixed time points (12, 24, 48 and 72 h), a few microliters were withdrawn to determine the released protein content by means of the colorimetric BCA test [43,67]. Thus, the degradation resistance of the SIS patch, DHT and DHT/EDC films was evaluated as the remaining weight % (*W_R_*) using the following equation:

(3) $$W_{R}\;\%=\left(1-\frac{W_{0}-W_{t}}{W_{0}}\right)\;\cdot \;100$$

where *W_t_* is the residual mass of the sample at time t and *W_0_* is the initial mass. Moreover, a single exponential decay rule (Equation (3)), reported for enzymatic degradation, was used to determine the in vitro half-life of the substrates, that is, the time t_1/2_ at which *W_t_/W_0_* = 0.5 [50,63].

(4) $$\frac{W_{t}}{W_{0}}=e^{-kt}$$

where *k* is a degradation rate constant (h^−1^). The test was performed in triplicate for each sample type and time point.

### 2.9. Surface Properties

Static contact angle measurement was performed dropping 10 μL milli-Q water on 10 × 10 mm samples using the sessile-drop method using a FTA1000 analysis system (First Ten Angstroms, Newark, NJ, USA) [68]. An average of three drops was carried out for each sample type.

The effect of the crosslinking strategy on the substrate surface topography and on the collagen native structure was evaluated by means of AFM. In particular, the experiments were performed using a Bioscope Catalyst scanning probe microscope (Bruker Inc., Billerica, MA, USA) mounted on a Zeiss Observer Z1 inverted optical microscope (Zeiss, Jena, Germany). The whole system was allocated on an insulating base that minimized the effects on measurements of environmental mechanical vibrations. The SNL V-shaped Bruker’s Sharp Microlever (Bruker Inc., Billerica, MA, USA), consisting in high sensitivity Silicon Nitride cantilever, was used to map the sample surfaces.

For each sample, the acquisitions were obtained on different scan areas (50 µm × 50 µm, 10 µm × 10 µm) at high resolution (512 × 512). The topographic images were analyzed by NanoScope Analysis software (Bruker Inc., Billerica, MA, USA) in order to quantify roughness parameters, which was expressed as R_q_:

(5) $$R_{RMS}=\sqrt{\frac{1}{n}\overset{n}{\underset{i=1}{\sum }}z_{i}^{2}}$$

where *n* is the number of data points and *z_i_* is the height deviation of *i*-th point from a mean line—defined so that the arithmetic sum of all *z_i_* is equal to zero. In detail, R_q_ was calculated as root mean square of z-channel variations with respect to mean height value calculated on whole scanned area, previously digitally modified by a second order plane fit and flattening in order to reduce the effects of bow and tridimensionality of sample. With the aim of achieving a more accurate local roughness value, R_q_ was calculated as mean value of twenty different areas of 1 µm × 1 µm.

The obtained data were analyzed and graphed using the OriginPro software (OriginLab version 8, Northampton, MA, USA) and the analysis of variance (ANOVA test) was used to establish the statistical significance of results.

### 2.10. Mechanical Properties

The mechanical properties of the proposed substrates were evaluated in hydrated state using a ZwickiLine universal testing machine (Zwick/Roell, Ulm, Germany) equipped with a loading cell of 1 kN by tensile test and suture redemption test.

For constitutive bond determination, samples of SIS patch, DHT and DHT/EDC films, sized at 5 × 20 mm, were firstly hydrated in 0.01 M PBS at room temperature for 1 h. Then, the thickness and width of wet specimens was measured using a Dino-Lite digital microscope (AnMo Electronics Corporation, New Taipei City, Taiwan). Tensile tests were performed under displacement-control till failure with a preload of 0.1 N and a load speed of 0.1 mm/s [68,69]. The Young modulus I, the stress at break (*σ*_max_) and the strain at break (*ε*_r_) of the samples were measured. In particular, E was calculated as the slope of the linear elastic region of the stress–strain curve at low strain values (in the range 1–5%). The experiment was performed in triplicate for each sample type.

The suture retention test was performed to determine the force necessary to pull a suture from the substrate or cause the wall of the graft to fail. In accordance with the standard practice ISO 7198, an absorbable 5–0 Coated VICRYL Polyglactin 910 (Ethicon, Johnson & Johnson International, New Brunswick, NJ, USA) suture was inserted at a distance of 2 mm from the edge of the hydrated specimens (0.01 M PBS, 1 h) to form a half loop [70]. The test was performed under displacement control with a universal testing machine ZwickiLine (Zwick/Roell, Ulm, Germany) equipped with a loading cell of 1 kN. A displacement rate of 150 mm/min up was set to cause the break of the sample. The suture retention strength was calculated as the maximum stress value recorded [71]. Each sample type was tested in triplicate.

### 2.11. In Vitro Response: Cell Culture and Immunostaining

Adult renal stem/progenitor cells (ARPCs) were isolated and characterized as previously described [72,73,74,75]. Cells were seeded on DHT, DHT/EDC films and SIS patch at a density of 5 × 10^4^ cells in their recommended medium, EGM (Lonza) with 20% of fetal bovine serum (Sigma-Aldrich, St. Louis, MO, USA). After cell expansion, stem cells were checked by immunofluorescence experiments by using as primary antibodies the mouse anti-human β-Actin mAb and the mouse anti-human CD133/2 mAb (clone AC133). The secondary antibodies Alexa Fluor 488 goat anti-mouse IgG and Alexa Fluor 555 goat anti-mouse IgG were used for β-Actin and CD133 staining, respectively. Nuclei were counter stained with DAPI (blue). The stained cells were viewed under the Leica TCS SP2 (Leica, Wetzlar, Germany) confocal laser-scanning microscope using ×40 and ×63 objective lenses.

The MTT viability assay (Sigma-Aldrich, St. Louis, MO, USA) was performed to determine cell growth following the supplier instruction. Briefly, cells were seeded in 48 wells at a density of 1.5 × 10^5^ cells for wells in EGM medium (Lonza) with 10% fetal bovine serum for 72 h in incubator at 37 °C, 5% CO_2_. Then, 10% MTT solution was added to each well. After an incubation of 4 h at 37 °C, MTT solvent was added to each well. After the formazan crystals had dissolved, triplicate aliquots were transferred to a 96-well standard plate. The 96-well plate was read by an enzyme-linked immunosorbent assay (ELISA) reader at 540 nm.

### 2.12. Statistical Analysis

All data were expressed as mean ± the standard deviation. Statistical significance of all experimental data was determined using *t*-Student except that of the AFM data, for which ANOVA test was used. Differences were considered significant at *p* < 0.05.

## 3. Results

### 3.1. Composition/Purity

**Figure 1:**
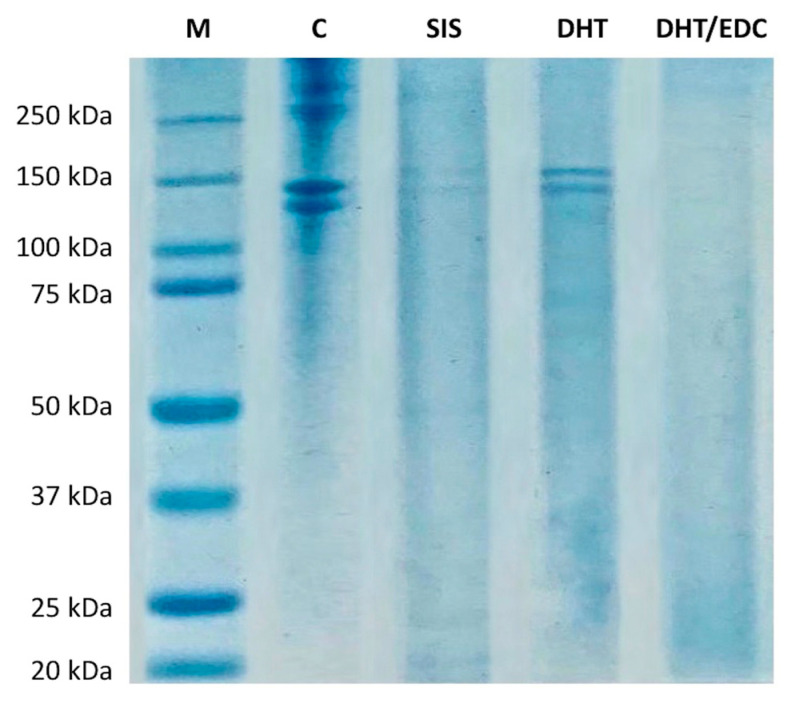
Electrophoresis patterns of DHT, DHT/EDC films and SIS patch in comparison with type I collagen gel (C) and standard proteins (M) with broad range of 20–250 kDa.

The SDS-PAGE was performed to investigate the protein composition of the SIS patch and the effect of the adopted crosslinking strategies on the collagen native structure of collagen-based prototypes. The electrophoretic pattern of SIS patch, DHT and DHT/EDC films in comparison with standard proteins is reported in Figure 1.

The protein pattern of type I collagen gel was provided as an example of the degree of integrity and purity of the material employed for the synthesis of the substrates under investigation [31]. As expected, the native collagen pattern was characterized by the presence of the typical two bands of type I collagen attributable to α1(I) chain of approximately 140 kDa and α2(I) chain of approximately 120 kDa, as confirmed by Western blot and MALDI/MS analysis performed in our previous work [31,54,55].

The protein pattern of DHT film confirmed that the air-drying technique and the DHT treatment did not affect collagen integrity, since no bands at low molecular weights were revealed. While collagen bands were clearly visible in the DHT film, no protein bands were detectable in the DHT/EDC film. The absence of collagen bands could be ascribed to the chemical EDC crosslinking that, by inducing zero-length crosslinks between the carboxylate moiety of aspartate and glutamate and amine residues of lysine, leads to the formation of high molecular weight aggregates that do not pass through polyacrylamide gel meshes [76,77]. Therefore, we assume that in our experiment, collagen subunits were highly crosslinked rather than degraded.

The SIS patch was also investigated in order to assess its protein composition. The electrophoretic pattern of the decellularized matrix is reported in Figure 1 and is characterized by the presence of two protein bands of approximately 140 kDa and 130 kDa. The SIS patch is known to be composed mainly of type I collagen (approx. 90%) [78,79,80] and, to a minor extent, of glycosaminoglycans (7%) [81], fibronectin (0.08%) [82] and collagen types III, IV and VI [78,83]. Since the sample preparation protocol applied allows detection of only the predominant protein component (fibronectin and GAGs require specific isolation protocols), the two high molecular bands on the SIS patch electrophoretic pattern could be ascribed only to type I collagen. The bands’ low intensity was instead due to the low extent of solubilized protein [84,85]. The decellularization and sterilization processes result in a more cross-linked and stiffer matrix compared to the native tissue that makes protein solubilization in the sample buffer more difficult [84,85]. Thus, the lower solubilized protein content in the sample buffer makes SIS patch bands less visible on the polyacrylamide gel.

### 3.2. Crosslinking Degree

The TNBS assay was performed to indirectly determine the extent of the crosslinking treatment. Physical or chemical crosslinking consists in the formation of bonds between the -NH_2_ and -COOH groups of amino acid residues of the collagen helices. Consequently, less free -NH_2_ groups are detectable in crosslinked matrices. With regard to the DHT film, it is evident that physical DHT treatment is able to reduce the number of free amino groups by approximately 20% (78 ± 2%, *p* < 0.02) through the formation of inter-chain hydrogen bonds in comparison with uncrosslinked film (100%) [48]. As expected, the addition of another crosslinking treatment by means of a chemical compound (EDC) allows the creation of more bonds, and thus a reduction in the number of free amino groups by a further 20% (58 ± 7%, *p* < 0.05).

The stress-relaxation tensile test was performed to measure the shear modulus and determine the elastically effective crosslinking density of the SIS patch, DHT and DHT/EDC samples, in accordance with the rubber elasticity theory (Table 1). As expected, the additional EDC crosslinking treatment on DHT film was found to have a significant effect on G, which increased 7-fold from approximately 0.08 kPa to 0.60 kPa (*p* = 0.002). The formation of additional bonds was also confirmed by the calculation of the crosslinking density, which increased from 4.8 × 10^−4^ mol/cm^3^ to 17 × 10^−4^ mol/cm^3^ (*p* = 0.006), according to the reduction in the free amino group content. With regard to the SIS patch, G was found to be nearly three times higher than DHT film (*p* = 0.002) and three times lower than DHT/EDC film (*p* = 0.006). Consequently, the crosslinking density of the SIS patch was found to be almost double that of the DHT film (*p* = 0.006) and the half of DHT/EDC film (*p* = 0.02).

### 3.3. Secondary Structure

**Figure 2:**
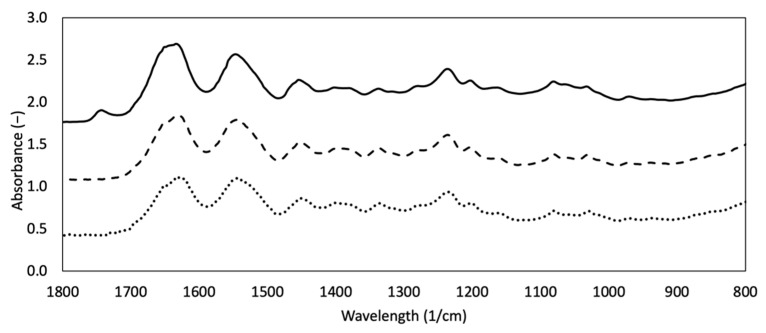
FTIR spectra of DHT (dotted line) and DHT/EDC (dashed line) substrates in comparison with the SIS patch (continuous line).

Spectroscopy is a powerful analytical technique to investigate biological samples. In particular, FTIR has always been used to acquire structural information about protein secondary structure and exposed functional groups on the surface of ECM. Native type I collagen has a peculiar IR spectrum characterized by three main peaks attributable to amide I, amide II and amide III, which are considered as the marker bands for collagen [24]. FT-IR spectra of DHT, DHT/EDC and SIS patch are reported in Figure 2. All samples are characterized by the three typical collagen signals. The amide I (1621–1635 cm^−1^) band, associated with C=O hydrogen bonded stretching, was recorded at 1635 cm^−1^ for DHT, 1621 cm^−1^ for DHT/EDC and 1634 cm^−1^ for SIS patch [43,64,86,87,88]. The amide II (1535–1548 cm^−1^) peak, that is associated with C-N stretching and N-H in plane bending from amide linkages, including wagging vibrations of CH_2_ groups from the glycine backbone and proline side-chains, was found 1548 cm^−1^ for DHT, 1538 cm^−1^ for DHT/EDC and 1547 cm^−1^ for SIS patch [43,64,86,87,88]. Finally, the amide III band, mainly ascribed to the N-H bending, was identified at 1243 cm^−1^ for DHT, 1227 cm^−1^ for DHT/EDC and 1236 cm^−1^ for SIS patch [43,64,86,87,88]. Bands at 1450, 1400 and 1340 cm^−1^ are assigned to the wagging and deformation modes of CH_3_ and CH_2_ of the glycine backbone and proline and hydroxyproline sides [64]. Bands of approximately 1080^−1^ and 1030 cm^−1^ are attributed to the stretching vibration of C-O-C and C-O, respectively [64].

The SIS patch spectrum confirmed that it is mainly constituted of type I collagen and that decellularization does not significantly affect collagen native structure. With regard to the collagen-based film spectra, the absence of significant shift in amide bands suggested that protein denaturation did not occur after the performed treatments (i.e., thermal crosslinking, chemical crosslinking). The 1230/1450 cm^−1^ absorption ratios were indeed in the range 0.8–1.0, confirming the integrity of the secondary structure of the protein triple helix. Interestingly, the shift to lower frequencies in the amides I and III of DHT/EDC suggested that C=O and N-H are probably involved in new bonding interactions, confirming the efficacy of the additional chemical crosslinking treatment.

### 3.4. Swelling Degree

**Figure 3:**
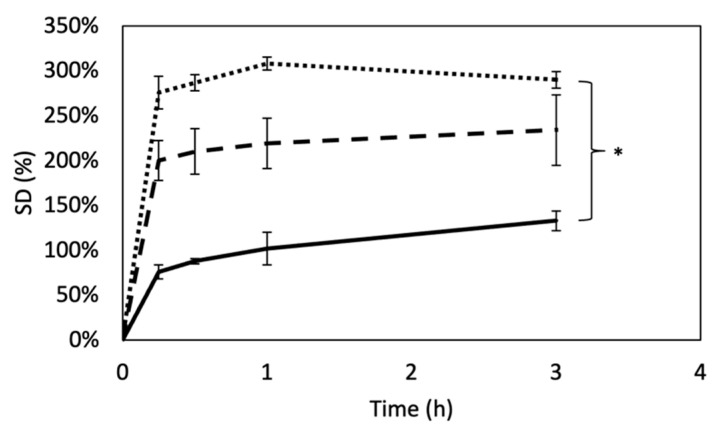
Swelling degree curves of DHT (dotted line), DHT/EDC (dashed line) and SIS patch (continuous line). Values indicated represent mean ± SD, where *n* = 3. Statistical analysis: Student’s *t*-test (* *p* < 0.01).

The ability of a scaffold to swell is an important aspect in tissue engineering, strongly related to the crosslinking degree. Swelling is known to play an important role in the mass transport [89]. The swelling ratio of DHT and DHT/EDC substrates in comparison with SIS patch is shown in Figure 3. As expected, data clearly shows that SD was significantly influenced by the crosslinking degree. The ability of DHT film to swell was indeed significantly reduced by the additional chemical crosslink with EDC (*p* = 0.01). However, the SD in comparison to SIS patch, a decellularized matrix that preserves all natural crosslinks among ECM components, was triplicated in the case of DHT (*p* = 0.00002) and duplicated in the case of DHT/EDC film (*p* = 0.001). These results were in partial disagreement with the elastically effective crosslink density values, for which a higher swelling degree for SIS patch was expected.

### 3.5. Thermal Properties

**Figure 4:**
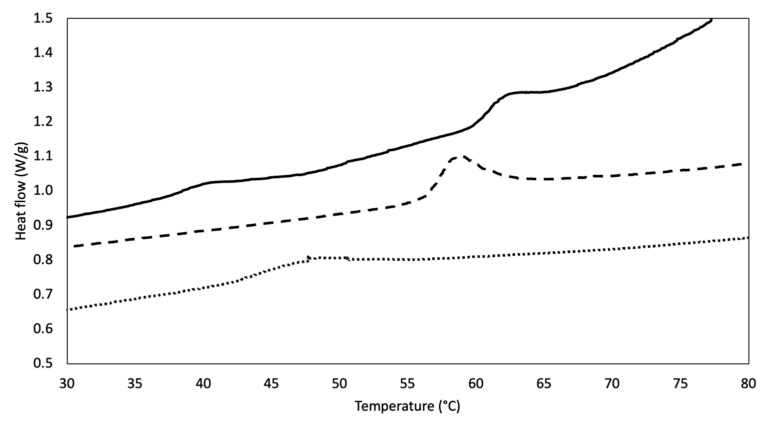
DSC thermograms representative of DHT (dotted line) and DHT/EDC (dashed line) substrates in comparison with the SIS patch (continuous line).

Calorimetric analysis by means of DSC allows assessment of substrate thermal behavior [90]. Representative thermograms of DHT and DHT/EDC prototypes in comparison with SIS patch are shown in Figure 4. Both collagen-based substrates showed an endothermic peak upon collagen denaturation temperature (43 °C) [31], a sign of matrix stability increment because of the crosslinking treatments. The DHT film exhibited an endergonic phenomenon at approximately 47.5 ± 0.2 °C. The application of the EDC crosslinking treatment on DHT film increased film stability, as confirmed by the increased denaturation temperature value, which shifted to 58.1 ± 1.1 °C (*p* = 0.006).

The thermal behavior of the SIS patch was also investigated and revealed the presence of two peaks, the first at approximately 35–45 °C and the second at approximately 60–70 °C. The detection of two phenomena could be attributed to the fact that the SIS patch is not only made of type I collagen (approx. 90%) but is also comprised, to a minor extent, of other ECM components, such as elastin, glycosaminoglycans and collagen types III, IV and VI [78,83]. Whereas the denaturation temperatures of some ECM components are similar to that of type I collagen (i.e., Td-GAG = 60 °C [91], Td-typeIIIcollagen = 40 °C [92]), with the exception of elastin (Td-elastin = 200 °C [93]), it is difficult to clearly attribute the first endothermic phenomenon to a specific ECM component. However, the second phenomenon, at approximately 62.4 ± 0.1 °C, may be attributable to the naturally crosslinked collagenous component, that is, the prevailing one. The contribution of the other minor components may be disregarded. Thus, the thermal stability of DHT/EDC film was comparable to that of the SIS patch, since there were no statistical differences (*p* = 0.03).

### 3.6. Degradation Resistance

**Figure 5:**
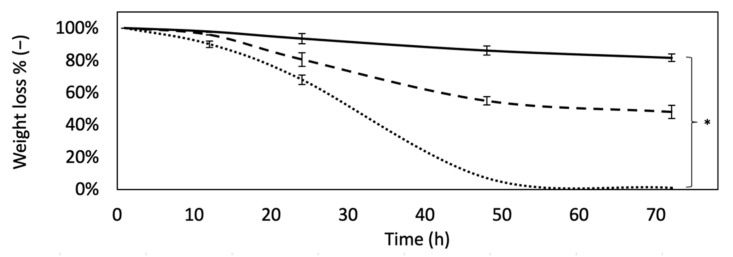
Degradation curves of DHT (dotted line), DHT/EDC (dashed line) and SIS patch (continuous line) obtained as film remaining weight (%), after different time points. Values indicated represent mean ± SD, where *n* = 3. Statistical analysis: Student’s *t*-test (* *p* < 0.001).

The scaffold’s resistance to enzymatic degradation was performed to simulate an accelerated in vivo proteolytic attack and investigate on their degradation kinetics. Figure 5 compares the degree of degradation over time of the DHT and DHT/EDC films with that of the SIS patch. While DHT film was visibly more sensible to degradation, DHT/EDC film and the SIS patch were revealed to be more resistant, with a remaining weight of approximately 50% and 80%, respectively, up to 3 days (*p* = 0.0001). The higher enzymatic resistance of the SIS patch could be ascribed to the fact that being a decellularized tissue, it retains all natural bonds that are more difficult to disrupt, resulting in less weight loss with a lower crosslinking density value. The mean degradation curve of the samples was fitted with an exponential rule generally applied to estimate samples in vitro half-life (Equation (4)). With regard to DHT film, t½ was determined to be approximately 28 h, while for DHT/EDC film it was found to be approximately 74 h (≈3 days), a value three times lower than that of the SIS patch, which was approximately 230 h (≈9 days).

### 3.7. Surface Properties

Cell-material interaction is considerably affected by surface properties. It is known that a moderate wettability is optimal for cell adhesion and proliferation [94]. The contact angle values instantly recorded on DHT and DHT/EDC substrates, being 87 ± 2° and 99 ± 3°, respectively, were comparable with the SIS patch (85 ± 6°) with no statistical differences (*p* = 0.50 and *p* = 0.06, respectively). The additional chemical crosslinking on DHT film did not significantly increase the hydrophobicity of the DHT/EDC substrate (*p* = 0.07). The formation of new bonding brought about a reduction in the number of chemical groups available for interaction with water molecules, which was reflected in slightly more hydrophobic behavior.

**Figure 6:**
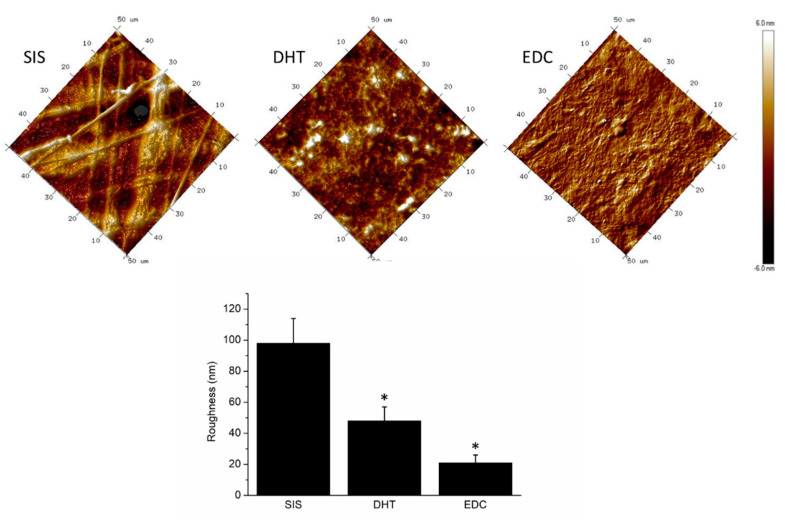
Representative AFM topographical acquisitions of different DHT and DHT/EDC substrates in comparison with SIS patch. In the histogram, the roughness (R_q_) values were reported as mean value with their standard deviations. Statistical analysis: ANOVA test (* *p* < 0.001).

The AFM was used to explore and compare the surface roughness of proposed substrates to that of the SIS patch. Moreover, a particular focus was given to surface modification induced by the adopted crosslinking strategies. In Figure 6, acquired scans revealed a fibrous structure for the SIS patch and a moderately rough surface for DHT and DHT/EDC films. The surface roughness, quantified in term of Rq, of the collagen-based substrates was found to be significantly different (ANOVA, *p* < 0.001) from that of the SIS patch, which was approximately 98 ± 16 nm. The collagen-based substrates were instead revealed to have an almost homogeneous rough surface, with an Rq of approximately 48 ± 15 nm for DHT and 21 ± 14 nm for DHT/EDC films. The superficial roughness of collagen-based substrates could be ascribed to the employed synthesis technique, which obtains steady and homogeneous surfaces with low roughness values. However, crosslinking treatments are known to strongly induce surface modifications [95]. As expected, the halving of DHT/EDC substrate roughness was ascribed to the chemical EDC treatment that, by coupling carboxylic and amine groups to yield amide bonds, turned out to be able to significantly reduce the roughness of the DHT/EDC film surface [95].

**Figure 7:**
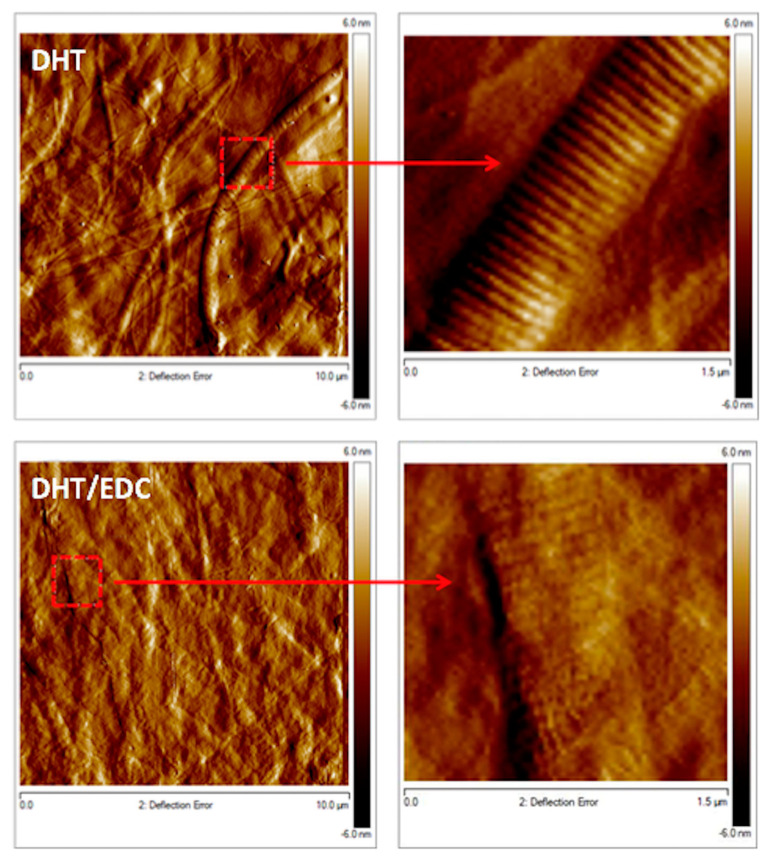
Representative topographic acquisitions of DHT and DHT/EDC substrates. On the right, images show the digital zoom on single collagen fibers.

In Figure 7, acquisitions at higher magnifications revealed the presence of long and randomly oriented collagen fibrils for both DHT and DHT/EDC films, confirming that the zero-length crosslinking treatments applied did not alter type I collagen native structure. Moreover, with a focus on D-banding pattern, a slight and insignificant change in fibrils D-period emerged (DHT: 61 ± 10 nm; DHT/EDC: 57 ± 14 nm, *p* > 0.05), confirming that the synthesis technique and the crosslinking strategies adopted not only did not alter the fibrillar structure of collagen, but did not induce significant shifts in the collagen natural banding of approximately 67 nm [31].

### 3.8. Mechanical Properties

**Figure 8:**
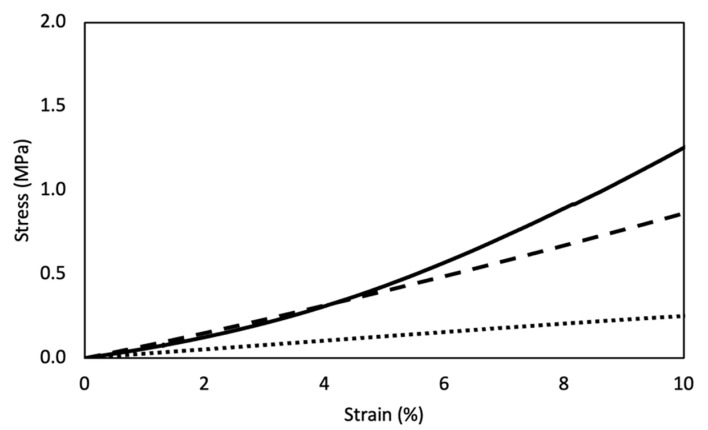
Representative stress–strain curves of DHT (dotted line), DHT/EDC (dashed line) substrates compared to SIS patch (continuous line).

A tensile test was performed to evaluate the mechanical properties of DHT and DHT/EDC films in the wet state, in comparison with the SIS patch (Figure 8). Tensile curves were characterized by a linear elastic region, followed by a non-elastic region and a rupture region. With regard to collagen-based films, while E (1–10%) was greatly increased from 3 MPa (DHT) to 8 MPa (DHT/EDC) (*p* = 0.00004) due to the additional chemical crosslinking, *σ*_max_ and *ε*_r_ were not significantly changed (*p* = 0.13 and *p* = 0.60, respectively) (Table 1). The constitutive bond of the SIS patch was found to be not statistically different from those of the DHT/EDC films in terms of E (*p* = 0.7) and *ε*_r_ (*p* = 0.9), while it differed in *σ*_max_ value, being almost doubled (*p* = 0.0007). Otherwise, the SIS patch showed different behavior not with regard to E and *ε*_r_, but in terms of linear zone extend (1–5%) and *σ*_max_. Although it is mainly composed of type I collagen, the smaller extent of the elastic region may be due to an alteration of the reticular structure of the SIS patch ECM after the decellularization and/or the sterilization processes.

The suturability of the proposed substrates was not a negligible parameter. The suturability values of the DHT and DHT/EDC films were very low when compared to that of the SIS patch, which, because of its natural origin, was strongly resistant to breakage (Table 1). The SIS patch is a decellularized matrix that retains in its structure all natural bonds between the ECM components, which give it great suture retention. On the other hand, the DHT and DHT/EDC substrates are only composed of a reconstituted type I collagen isolated from equine tendon and reorganized in a sheet-like form. However, despite the low suture retention values of both DHT and DHT/EDC films compared to the SIS patch (*p* = 0.0004 and *p* = 0.0003, respectively), these substrates are able to resist suturing since, for the best of our knowledge, a prototype with a much lower value was successfully implanted in a rabbit model in vivo [20].

### 3.9. Biological Evaluation

**Figure 9:**
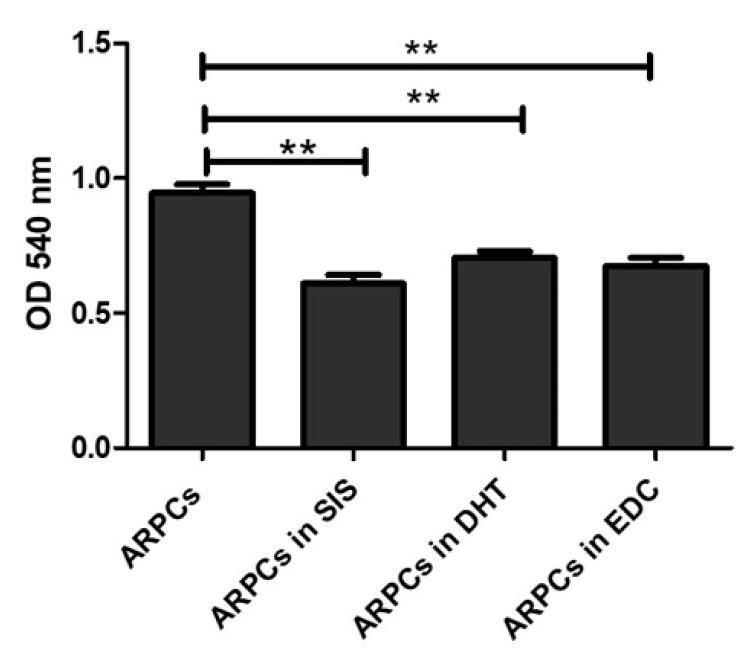
MTT assay showing the viability of cells in culture on DHT, DHT/EDC and SIS films. (** *p* value < 0.005).

In order to study both the biocompatibility of the proposed materials and their suitability as substrates for urethral regeneration, ARPCs were cultured on the SIS, DHT and DHT/EDC substrates for 7, 14, 21 and 28 days. First of all, cell proliferation and viability were quantitatively evaluated in the three kinds of films at 72 h. The MTT assay was used to measure cellular metabolic activity as an indicator of cell viability, proliferation and cytotoxicity. As reported in Figure 9, no significant differences in ARPC growth on DHT and DHT/EDC films compared to the SIS patch was observed.

**Figure 10:**
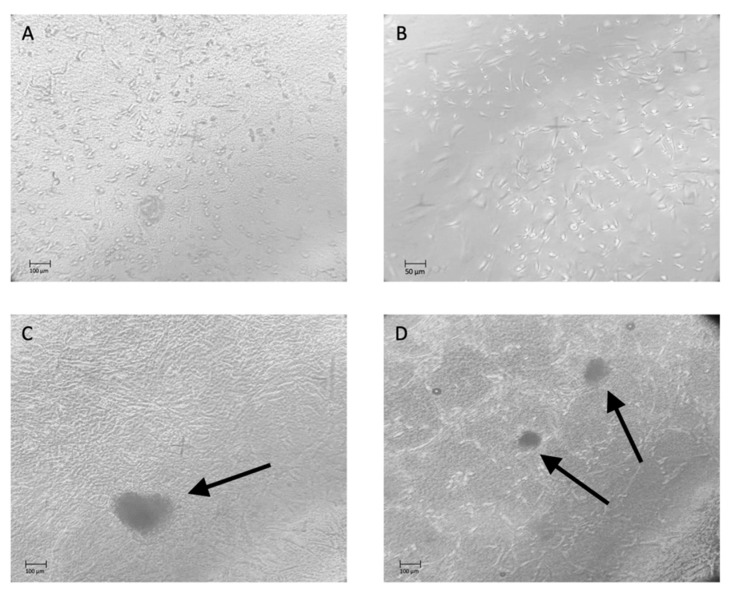
Phase-contrast microscopy images showing ARPCs growing in monolayer after 14 days of culture on DHT (**A**) and DHT/EDC films (**B**). After 21 days of culture, spheroids appeared in culture, composed of ARPCs seeded on DHT/EDC (**C**), and after 28 days spheroids and renal tubular like structures were observed (**D**). Original Magnification: 20×.

After 14 days of culture, ARPCs formed a monolayer on the DHT and DHT/EDC substrates (Figure 10A,B, respectively). After 21 days, ARPCs were able to organize themselves into 3D structures known as spheroids, which were functional for the regenerative processes (Figure 10C). After 28 days of culture on DHT/EDC a more numerous presence of spheroids and the formation of renal tubular-like structures were observed (Figure 10D).

In order to explore the viability and growth of ARPCs on films, cells were stained for the cytoplasmatic marker β-actin and the surface marker CD133. β-Actin is a highly conserved, non-muscle cytoskeleton protein involved in cell motility, structure and integrity, and therefore it is useful for monitoring changes in the cell shape. The CD133 is a marker typical of ARPCs that is used for their isolation. In addition, it is an inverse marker of renal stem cell senescence and a direct marker of renal stem cell function. For these reasons, monitoring the CD133 marker allows checking of ARPC viability and stemness.

**Figure 11:**
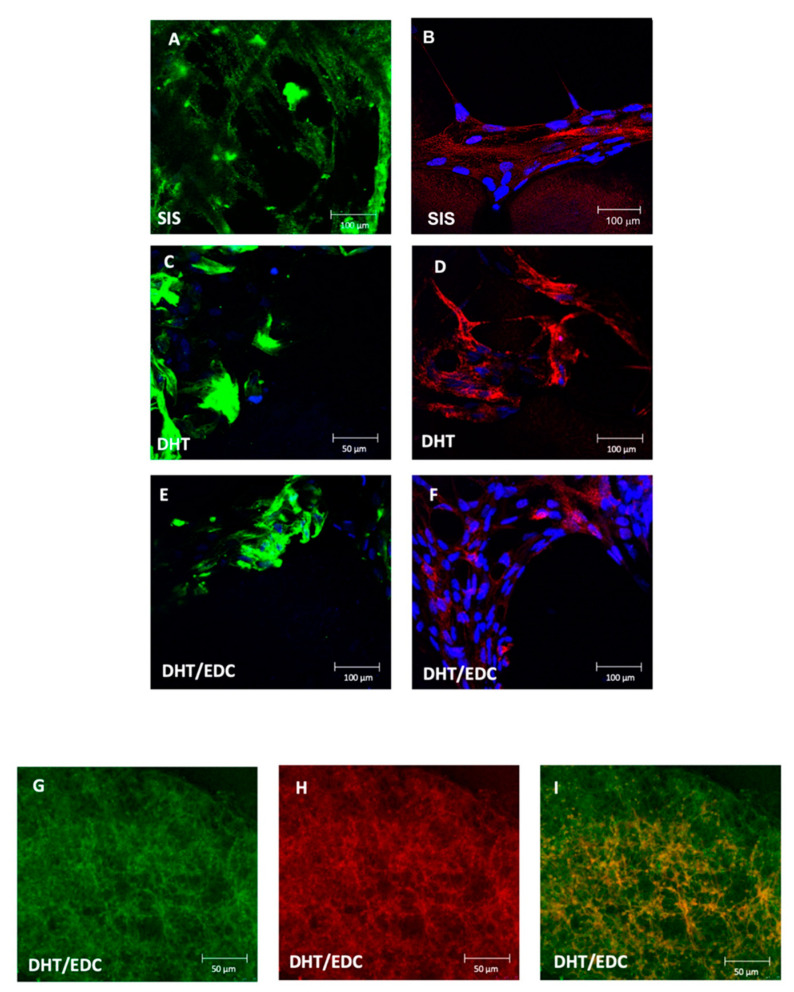
Immunofluorescence experiments on ARPCs showing a different pattern of growth on DHT and DHT/EDC films compared to SIS patch. Stem cells were stained for β-Actin (green) and CD133 (red); nuclei were counterstained with DAPI (blue). Original Magnification: 63×. Panels (**A**,**B**) represent ARPCs cultured on a SIS patch and stained for β-Actin (green, (**A**)) and CD133 (red, (**B**)). Panels (**C**,**D**) represent ARPCs cultured on DHT film and stained for β-Actin (green, (**C**)) and CD133 (red, (**D**)). Panels (**E**,**F**) represent ARPCs cultured on DHT/EDC film and stained for β-Actin (green, (**C**)) and CD133 (red, (**D**)). Panels (**G**–**I**) represent spheroids generated from DHT/EDC films and stained for β-Actin (green, (**G**)) and CD133 (red, (**H**)). Panel (**I**) represents the overlay between the β-Actin and CD133 staining.

The growth capacity of cells cultured on DHT and DHT/EDC films was compared to that of SIS patch. As shown in Figure 11, all substrates were biocompatible and able to sustain cell growth. From a morphological point of view, both in collagen-based films and the SIS patch, ARPCs formed actin stress fibers, typical of cellular contractility, providing force for a number of functions, such as cell adhesion, migration and morphogenesis. Actin stress fibers were more evident in cells cultured on SIS patch (Figure 11A), on which cells were also more elongated compared to cells in culture on DHT film (Figure 11C) and DHT/EDC film (Figure 11D). However, ARPCs were found to populate the collagen-based supports more steadily than the SIS patch. In particular, the DHT/EDC film allowed a faster growth, so that cells reached an 80–90% confluence in 14 days. Conversely, cells cultured on SIS patch slowly reached 80% confluence at 21 days (data not shown), confirming the low effectiveness of this decellularized matrix for urethral tissue regeneration widely reported in literature [96,97,98].

In addition, the stemness of ARPCs on collagen-based substrates was investigated. It was found that at the 28th day cells preserved the CD133 marker both on the DHT and DHT/EDC films (Figure 11E,F). However, differences were present between the DHT and DHT/EDC films, since cells cultured on DHT/EDC film formed spheroids (Figure 11G–I), while this phenomenon was not observed on DHT film, where ARPCs were found to dig some niches inside the collagen matrix. Thus, the application of the EDC crosslinking on the DHT treated film, by increasing the DHT/EDC substrate crosslinking degree, increases also its stiffness and degradation resistance, recreating a condition favorable for the formation of spheroids. The phenomenon observed on DHT film instead was attributed to the lower crosslinking degree, which makes it more susceptible to proteolytic and less stiff, with a Young modulus three times lower than DHT/EDC film and SIS patch. The rapid digestion of the substrate should not be a favorable condition, since the rapid loss of mechanical performance would not be compliant with the regeneration time necessary to correctly support and sustain the regenerative process until the formation of a new tissue.

These results accord with the well-known effect of crosslinkers on the GFOGER integrins binding sites of collagen helical structure: the hiding or the removal of such recognition sites is reported to significantly alter cell processes, since cell response is mediated by integrin bonding in a mechanosensing and ligand density-dependent way, and to direct them towards different pathways [99].

## 4. Discussion

Urethral stricture consists in the abnormal fibrosis of the epithelial tissue and corpus spongiosum, resulting in the narrowing of the urethral lumen [13]. This pathological condition adversely impacts not only patients’ quality of life, but also on their overall health status. Current surgical strategies are not resolutive, since a certain degree of stricture recurrence rate is registered with all techniques within 2–5 years of follow up. Beyond these strategies, the gold standard is urethroplasty, an augmentation treatment with a long-term success rate significantly better than dilation or any endoscopic treatment [100]. Urethroplasty is performed with autografts (i.e., buccal mucosa) or decellularized matrix (i.e., SIS patch). The urethral augmentation with autologous tissue requires staged surgery, additional expertise in oral cavity surgery of the urologist or the aid of an oral surgeon in order to correctly sever the tissue and avoid post-operative discomfort. Besides the numerous donor site morbidities (i.e., pain, infection, discomfort, numbness, speech problems, impairment of mouth opening and damage to salivary ducts [10,15,101], buccal mucosa collection is hindered in cases of ongoing oral infection, restricted mouth opening or extension and previous mouth or tongue surgery [16]. Thus, issues related to buccal mucosa availability and post-operative discomforts, together with the requirement of a double surgery mean autografts are not always prosecutable.

On the other side, the SIS patch mediated augmentation avoids all aforementioned issues. Thus, the use of a device to be implanted could be considered a more convenient solution for the surgeon and a less invasive treatment for the patient. However, urethroplasty by means of SIS patch is not resolutive, since it represents a 20–25% stricture recurrence rate within 1–5 years of follow-up [16].

In this circumstance, tissue engineering could offer new strategies to treat some unresolved diseases of the urinary tract, such as urethral strictures, by means of TEMPs. Indeed, autologous cell-seeded scaffolds were recently considered as the better strategy to reach good regeneration levels [102,103]. Even though the presence of cells has a strong impact on tissue regeneration time and quality, the biomaterial and the manufacture process have significant influence on cell response. For this purpose, several kinds of biomaterials were employed [104] but, among them, collagen-based formulations always gave back best in vitro and in vivo results [18,19,102].

In this work, with the ultimate purpose to manufacture a substrate to be autologous cell seeded prior to implant, two collagen-based substrates were designed to both provide mechanical properties compliant with urinary tract physiological stress, and to better sustain tissue regeneration compared to the SIS patch, the only commercially available and clinically approved device that actually represents the only alternative to autograft. In particular, DHT-treated and DHT/EDC-treated collagen-based substrates were proposed and characterized from a biochemical, physicochemical and biological point of view.

Firstly, since the preservation of collagen native structure after manufacturing is fundamental for cell response, variations in collagen molecular weight were investigated by means of SDS-PAGE. Moreover, SIS patch protein composition was investigated, revealing a protein content consisting mainly of type I collagen [78,83], since two high molecular bands attributable to type I collagen were principally present. As reference, type I collagen suspension employed for the substrate synthesis was analyzed, confirming the nativeness and integrity of the starting material due to the absence of non-collagenous bands [31]. The electrophoretic pattern of collagen-based substrates confirmed that the applied crosslinking treatments did not induce collagen degradation. In particular, while α1(I) and α2(I) bands were clearly visible in the DHT film, no protein bands or smear were detectable in the DHT/EDC film because of the formation of high-molecular-weight crosslinked aggregates that did not pass through the polyacrylamide gel meshes [76,77,105]. This result was supported by AFM analysis, in which type I collagen native fibrillar structure with the typical D-banding periodicity was clearly visible for both DHT and DHT/EDC films. Accordingly, the presence of unaffected type I collagen amide bands in all FT-IR spectra confirmed the preservation of the protein secondary structure. Thus, the preservation of collagen native structure confirmed that the synthesis technique and the crosslinking strategies adopted not only did not alter the native fibrillar structure of collagen, but did not induce significant shifts in the collagen natural banding [31].

The effectiveness of the crosslinking treatments was assessed by means of the free amino group content and the elastically effective crosslinking density. The physical dehydrothermal treatment applied was revealed to be able to reduce the number of free amino groups by approximately 20% through the formation of inter-chain hydrogen bonds [48]. The addition of EDC zero-length crosslinking reduced the number of free amino groups by a further 20%. Moreover, the calculation of the elastically effective crosslinking density by means of the application of the rubber theory allowed confirmation of the increase in the crosslinking density and, as a consequence, of G value. Accordingly, the amide I and III peaks shifting to lower frequencies suggested that the C=O and N-H groups of DHT/EDC were involved in new bonding interactions, confirming the efficacy of the additional chemical crosslinking treatment. In comparison, the SIS patch, although a naturally crosslinked matrix, turned out to be almost doubly crosslinked in comparison with DHT film and the half of DHT/EDC film. Likewise, the G value of the SIS patch was found to be nearly three times higher than DHT film and three times lower than DHT/EDC film. The effectiveness of the crosslinking treatments applied was also confirmed by swelling analysis and contact angle evaluation. The number of free groups is strictly related to the crosslinking degree and thus to the swelling behavior inasmuch as the capability of matrices to retain water depends on the number of interaction sites with water molecules. Thus, the collagen-based substrates’ ability to swell was significantly influenced by the crosslinking degree, with a reduction in DHT/EDC film swelling ability of approximately 20% compared to DHT film. The formation of bonds involves a decrease in the number of chemical groups available for interactions with water molecules, which reflects a slightly more hydrophobic behavior visible from the slight but not significant increase in the contact angle value. Otherwise, the SIS patch showed the lowest water retention ability and a surface affinity with water comparable to collagen-based films.

The effect of crosslinking on the surface morphology of proposed collagen-based substrates was thoroughly investigated by means of AFM analysis. Acquired scans revealed a moderately rough and homogeneous surface for collagen-based films, attributable to the employed synthesis technique that obtains consistent surfaces with low roughness values. The Rq halving in DHT/EDC film compared to DHT film was ascribed to the applied chemical crosslinking treatment [95]. In comparison, the SIS patch was revealed to be characterized by a fibrous and irregular structure with a higher roughness value.

As widely known, crosslinking treatments not only influence surface chemistry and physical–chemical properties, but have a strong impact also on substrate thermal properties and susceptibility to proteolysis. The application of the EDC crosslinking on DHT film, by inducing the formation of additional amide bonds [99], was revealed to be able to induce a shift in the collagen denaturation temperature that approached that of the SIS patch. Similarly, collagen-based substrate resistance to the in vitro proteolytic attack followed the same behavior: while DHT film was visibly more sensible to degradation, DHT/EDC film and the SIS patch followed the same trend, with an estimated half-life of approximately 3 days and 9 days, respectively.

Tensile tests were performed to verify whether the collagen sheets’ mechanical properties were compliant with this application. The constitutive bond of DHT/EDC film was found to be not statistically different from that of the SIS patch in terms of E and *ε*_r_, confirming that DHT/EDC film could be a promising substitute to the SIS patch. However, the E value of DHT/EDC film (8 MPa) was found to be higher than that of the collagen-based devices proposed for urethral regeneration, such as an uncross-linked high-density collagen tube (approximately 28 kPa) [20] or silk/keratin/gelatin film (65–107 kPa) [18]. Additionally, suture retention tests revealed strong enough seal values to ensure the substrate’s implantability, even if it was very low compared to the SIS patch. However, this aspect may not be limiting, since prototypes with much lower suture resistance value were successfully implanted in vivo [20].

The biochemical, physical–chemical and mechanical characterization allowed identification of the DHT/EDC substrates as the closest configuration to that of the SIS patch. However, our goal was not to produce a substitute identical to SIS patch, but to realize a substrate that sustains cell attachment and growth significantly better. Indeed, the choice of an air-dried bulk substrate relies on the fact that it should be impermeable to body fluids and support the urethral mucosa regeneration. Urothelial regeneration is therefore fundamental for the success of the implant, since it acts as a barrier that protects surrounding tissues from the caustic properties of urine. Thus, the rapid formation of a cell monolayer on the temporary scaffold is necessary.

The collagen-based substrate’ biocompatibility was verified by the seeding of ARPCs, a cell type able to differentiate in several kind of cells and to have reparative capacity in different damage settings [72,75,106]. Cells were cultured on collagen-based films and SIS patch and were allowed to grow for 7, 14, 21 and 28 days. After this period, cell morphology, evaluated by the assessment of the actin cytoskeleton, revealed that all substrates were biocompatible and able to sustain cell adhesion and grow, since actin stress fibers were clearly and immediately exhibited. Actin is a cytoplasmatic globular protein that plays a major role in different processes, such as migration, morphogenesis, cytokinesis, endocytosis and phagocytosis, and is crucial for many developmental and physiological processes in multicellular organisms [107]. Interestingly, a recent study demonstrated that matrix rigidity and cell shape regulate the polarization of stress-fibers of cytoskeleton in cells, and that the alignment of stress-fibers in stem cells depends on matrix rigidity, attaining a maximum value when the cell and matrix rigidity are similar [108]. In fact, stress fibers are involved in cell mechanotransduction, a process that implies the transduction of forces from the outside to the inside of the cell and that can control the maturation or disassembly of cellular adhesions, and initiate intracellular signaling cascades altering cellular behaviors [109]. These findings support the hypothesis that the environment has a great influence on the characteristics and behavior of stem cells. In this regard, DHT/EDC films were found to be able to promote cell growth and proliferation more easily than SIS patch and DHT film. Consequently, the 80–90% cell confluence was observed after 14 days on DHT/EDC films, and only after 21 days on SIS patch. The less favorable conditions offered by SIS patch, confirmed by the slower cell proliferation rate, were in accordance with the low effectiveness of this decellularized matrix for urethral tissues regeneration [96,97,98], for which restenosis is observed for 20–25% of cases [16].

Another critical point for a regeneration model based on stem cells is the maintaining of stemness and the formation of spheroids. After cell growth on different films, ARPCs were stained for CD133, a biomarker typical of ARPCs that is used for their isolation, and that is well known to be involved in regeneration and repair processes, in cell proliferation and in senescence [110,111]. In human kidney, CD24+/CD133+ cells in different nephron segments behave as a group of resident ARPCs, which have the ability, in vitro and in vivo, of expansion, self-renewal and epithelial differentiation [110]. The expression of CD133 is positively correlated with cells’ functional properties. It is therefore important that its expression remains high during cell growth to avoid uncontrolled cell differentiation. Here, immunofluorescence staining demonstrated how DHT/EDC film promoted the formation of ARPCs spheroids along with tubular-like structure and CD133 retention also after 28 days of culture. The formation of three-dimensional structures is of fundamental importance for cell process studies, since organoids better represent the physiological system than their two-dimensional culture counterparts for the purpose of organ regeneration studies, as they recapitulate the signaling and morphological cues that can occur within the human body. The spheroids indeed resemble the structure, physiology and diseases of their organ of origin, and are usually generated from either pluripotent stem cells (PSC) or adult stem or progenitor cells (ASC) [112]. These spheroids, which were shown to maintain a strong expression of both β-actin and CD133, can be maintained in culture for long periods of time. The proliferative capacity and the expression of CD133, which define stemness and dedifferentiation, indicated that the spheroids recapitulate the renal plasticity-based regeneration response in vitro, since the lack of CD133 impaired the generation of nephrospheres while favoring senescence [110,112].

These biological data support the hypothesis that the DHT/EDC film could be a suitable substrate for stem cell in vitro studies, and have the strong potential to be an ideal scaffolding material for the development of new and effective implantable devices for urethral regeneration, more than the SIS patch.

## 5. Conclusions

The unavailability of resolutive surgical treatments for urethral stenosis has attracted the interest of scientific research that, in the last decade, has tried to find new resolutive strategies. In this context, tissue engineering and regenerative medicine approaches offer the possibility to reach better outcomes with the use of implantable alternatives to autografts. As is known, urethral augmentation with autologous tissues has the best outcomes, but requires staged surgery, additional expertise in oral cavity surgery and involves post-operative discomforts. Alternatively, implantable devices avoid all the aforementioned issues and are considered more convenient and less invasive solutions. However, urethroplasty with decellularized tissues (i.e., SIS patch) represent high stricture recurrence rates. In this circumstance, with the ultimate goal of manufacturing a scaffolding material suitable for urethral regeneration with a reconstituted natural biomaterial, a thin DHT/EDC crosslinked collagen substrate was developed in order to be impermeable to cells and fluids, to support cells to grow better than with an SIS patch, and to resist the mechanical stress to which the urethra is physiologically subjected for the entire time-period required for tissue regeneration. The proposed collagen-based substrate was characterized from a biochemical, physicochemical and biological point of view in comparison with the SIS patch, the only commercially available and clinically approved xenograft that represents the only alternative to autograft. To the best of our knowledge, this work is also one of the few that clearly report an almost complete SIS patch characterization in terms of composition, physical-–chemical, mechanical and thermal properties, adding a significant contribution to the development of new devices for urethral regeneration. The preliminary testing of such substrates enabled identification of the optimized collagen formulation and crosslinking protocols to preserve the type I collagen native structure fundamental for cell processes, and enabled the realization of a substrate with appealing properties. In particular, the electrophoretic pattern and AFM imaging confirmed that the applied crosslinking treatments did not induce collagen degradation, since variations in collagen molecular weight were not detected, and its native fibrillar structure with the typical banding was clearly visible. The decrease in free amino group content and the increase in elastically effective crosslinking density confirmed the effectiveness of the crosslinking treatments. Accordingly, degradation resistance and thermal properties were found to be significantly improved. Mechanical tests confirmed collagen sheet compliance with this application in terms of constitutive bond, and suture retention that revealed a strong-enough seal value to ensure the substrate’s implantability.

Lastly, the collagen-based substrate biocompatibility was verified by the seeding of ARPCs, a cell type able to differentiate in several kind of cells and to have reparative capacity in different damage settings. Cell morphology and immunostaining revealed that substrates were biocompatible and able to sustain cell adhesion up to 28 days. In this regard, DHT/EDC films were found to be able to promote cell growth and proliferation more easily than SIS patch. Consequently, the 80–90% cell confluence was observed after 14 days on DHT/EDC films, and only after 21 days on SIS patch. In urothelial regeneration, the rapid formation of a cell monolayer on the temporary scaffold is fundamental for the success of the implant, since it acts as a barrier that protects surrounding tissues from the caustic properties of urine. The less favorable conditions offered by SIS patch, confirmed by the slower cell proliferation rate, were in accordance with the low effectiveness of this decellularized matrix, for which restenosis is usually observed.

Interestingly, DHT/EDC film also promoted the formation of ARPC spheroids along with tubular-like structures CD133+ after 28 days. The formation of three-dimensional structures is of fundamental importance for cell process studies, since organoids better represent the physiological system than their two-dimensional culture counterparts.

Thus, thanks to collagen nativeness preservation along with the presence of the right physical–chemical, biochemical and mechanical stimuli, the formations of a stem cell monolayer after only 14 days and spheroids after 21 days were obtained, confirming also the substrate biocompatibility and suitability to sustain cell processes better that SIS patch. The formation of an ARPC monolayer in 14 days offers the prospect that collagen-based substrate may be able to recreate the urothelium in a short time, therefore favoring regenerative processes to the detriment of fibrotic ones. The ARPCs spheroids, although they require extensive further investigations, could be the starting point for the identification and stabilization of in vitro 3D models, useful for accurate study of cell behavior in physiological and pathological states prior to device testing on in vivo models.

## Figures and Tables

**Table 1:** Properties of DHT and DHT/EDC films compared to SIS patch in terms of shear modulus (G), elastically effective crosslinking density (*ρ*), Young modulus (E), maximum stress (*σ*_max_), strain at break (*ε*_r_) and suture retention strength (SR). Values indicated represent mean ± SD, where *n* = 3.

Sample	G (kPa)	*ρ* (mol/cm^3^)	E (MPa)	*σ*_max_ (MPa)	*ε*_r_ (%)	SR (g)
**DHT**	0.08 ± 0.01	4.8 ± 0.3 × 10^−4^	2.9 ± 0.5	2.3 ± 1.2	55.6 ± 20.7	52 ± 7
**DHT + EDC**	0.60 ± 0.11	17.0 ± 4.1×10^−4^	8.3 ± 1.4	3.4 ± 1.0	39.1 ± 11.0	24 ± 3
**SIS**	0.23 ± 0.04	7.6 ± 0.9 × 10^−4^	8.8 ± 1.7	7.1 ± 0.8	40.8 ± 5.9	520 ± 35
